# Diagnostic utility of 3D MRI sequences in the assessment of central, recess and foraminal stenoses of the spine: a systematic review

**DOI:** 10.1007/s00256-024-04689-1

**Published:** 2024-04-27

**Authors:** Mika T. Nevalainen, Juho Vähä, Lasse Räsänen, Michaela K. Bode

**Affiliations:** 1 Faculty of Medicine, Research Unit of Health Sciences and Technology, University of Oulu, POB 5000, 90014 Oulu, Finland; 2 Department of Diagnostic Radiology, Oulu University Hospital, P.O. Box 50, 90029 Oulu, Finland

**Keywords:** Imaging three-dimensional, Magnetic resonance imaging, Spine, Spinal stenosis

## Abstract

**Objective:**

To perform a systematic literature review on the diagnostic utility of 3D MRI sequences in the assessment of central canal, recess and foraminal stenosis in the spine.

**Methods:**

The databases PubMed, MEDLINE (via OVID) and The Cochrane Central Register of Controlled Trials, were searched for studies that investigated the diagnostic use of 3D MRI to evaluate stenoses in various parts of the spine in humans. Three reviewers examined the literature and conducted systematic review according to PRISMA 2020 guidelines.

**Results:**

Thirty studies were retrieved from 2 595 publications for this systematic review. The overall diagnostic performance of 3D MRI outperformed the conventional 2D MRI with reported sensitivities ranging from 79 to 100% and specificities ranging from 86 to 100% regarding the evaluation of central, recess and foraminal stenoses. In general, high level of agreement (both intra- and interrater) regarding visibility and pathology on 3D sequences was reported. Studies show that well-optimized 3D sequences allow the use of higher spatial resolution, similar scan time and increased SNR and CNR when compared to corresponding 2D sequences. However, the benefit of 3D sequences is in the additional information provided by them and in the possibility to save total protocol scan times.

**Conclusion:**

The literature on the spine 3D MRI assessment of stenoses is heterogeneous with varying MRI protocols and diagnostic results. However, the 3D sequences offer similar or superior detection of stenoses with high reliability. Especially, the advantage of 3D MRI seems to be the better evaluation of recess stenoses.

## Introduction

In the routine imaging of the spine, conventional T1 and T2 -weighted and STIR MRI sequences in different planes – namely sagittal and axial – have been deemed as the gold standard diagnostic tool for decades. The MRI of the spine offers valuable information to the referring physician and is often the key to the modern treatment of various spinal disorders. Since the 2010s, the isotropic submillimeter 3D sequences have become steadily more available with different MRI devices – these thin-slice sequences offer superior resolution as compared to the conventional thick-slice (roughly 3–4 mm) MRI sequences. Fifteen years ago, the bottleneck of these techniques was the long imaging time as compared to the conventional sequences. Due to the development of MRI equipment, deep learning techniques, and imaging sequences, the modern 3D sequences no more pose this challenge. However, the adoption of the 3D sequences to the everyday clinical setup has been rather slow, and conventional thick-slice protocols are still widely in use. Accordingly, the purpose of this systematic review was to study the current status of 3D MRI of the spine related to evaluation of stenoses (intra/extraforaminal, recess and central canal).

## Materials and methods

A systematic literature search was performed with no restrictions on publication type or language within the following databases: PubMed, MEDLINE (via OVID) and The Cochrane Central Register of Controlled Trials, from the records published from January 1st 2000 to May 29th 2020, and updated on January 20th 2023. Search terms covered the following domains: spine, MR, MRI, thin-slice, three-dimensional, isotropic, 3D and high-resolution). Reference and citation tracking of included articles and related reviews within the topic was performed to detect further studies. The Preferred Reporting Items for Systematic reviews and Meta-Analyses (PRISMA) 2020 statement was used to guide the conduct and reporting of this study [1].

## Study selection

The articles returned from the search were screened using a two-stage process. Two reviewers (a fellowship-trained neuroradiologist with 20 years of experience and a fellowship-trained musculoskeletal radiologist with nine years of experience) screened titles and abstracts against the eligibility criteria in the first stage. In the second screening round, full-text versions of the potentially relevant studies were screened by the reviewers (roughly 50%-50%). When necessary, potentially interesting articles were discussed by the radiologists and a medical physicist involved in the review process at this stage. The primary eligibility criterion was the use of 3D MRI sequences in the assessment of spinal stenoses including foraminal, recess and central stenoses in 1.5 T or 3.0 T MRI devices on cervical, thoracic or lumbar spine. The secondary eligibility criterion was the comparison of 3D MRI to routine 2D MRI or to surgical findings; thus the gold standard for diagnosing stenosis varied between the reviewed studies. Reasons for exclusion of full-text articles were case reports, the lack of isotropic 3D MRI sequences (voxel resolution more than 1 mm) and lack of comparison between 2 and 3D sequences.

## Results

Our systematic search identified 2 595 papers. Ten additional studies were identified through previous reviews and citation tracking of included articles. After removing duplicates, and title and abstract screening, 62 records were deemed relevant for full-text screening. After the full-text screening, 30 papers were included in this study. Tables 1 and 2 summarize the relevant included articles, where the diagnostic utility of lumbar (Table 1) or cervical (Table 2) 3D MRI sequences were studied.

**Table 1:** Studies comparing 2D and 3D MRI sequences in lumbar spine

Authors	Publication year	Sample size (cases/controls)	Field strength and MRI machine	Inter ± intraobserver (yes/no)	MRI sequences used	Diagnostic performance (sens/spec etc.) (yes/no)	Surgical correlation (yes/no)
Nemoto et al. [6]	2014	15 patients, 10 healthy volunteers	1.5 T, GE Signa infinity Excite	yes	3D FIESTA (AX, SAG, COR recons) vs. 2D FSE (T1 and T2 SAG & AX)	yes	yes
Blizzard et al. [12]	2015	80 patients	1.5 T, Siemens Avanto and Esprit; 3.0 T, Siemens Verio	yes	3D T2 TSE vs 2D FSE (T1 & T2 AX & SAG and PD AX)	no	no
Sayah et al*.* [8]	2016	250 patients	1.5 T, Siemens Magnetom Avanto	no	3D T2 SPACE SAG vs. 2D T2 TSE (T1 and T2 SAG & AX) & STIR SAG	yes	no
Swami et al*.* [14]	2016	20 patients	1.5 T, Siemens (model not indicated)	yes	3D T2 SPACE SAG vs routine T2 and T1 SAG and AX images	no	no
Takashima et al*.* [25]	2016	20 healthy volunteers	1.5 T, GE Signa HDxt	no	T2 3D FIESTA vs. T2 3D COSMIC vs. T2 3D COSMIC FS (orientation not indicated)	no	no
Koontz et al. [9]	2017	118 patients	1.5 T, Siemens Aera and Avanto	yes	3D T2 SPACE FS vs. conventional LS MRI protocol	yes	no
Hossein et al*.* [11]	2018	35 patients	1.5 T, Siemens Magnetom Avanto	yes	3D T2 SPACE SAG vs 2D TSE (T2 SAG, T2 AX & T2 COR)	no	no
Kinoshita et al*.* [24]	2018	677 patients	1.5 T, Philips Intera Achieva Nova Dual R 2–6	no	3D DE-VISTA-AFI (T2 DE-VISTA COR + PD DE-VISTA COR) vs. 3D PD DE-VISTA COR, 3D T2 DE-VISTA COR and 2D T2 TSE AX	no	no
Hashimoto et al. [4]	2021	54 patients	1.5 T, Magnetom Avanto	yes	3D T1 FLASH, no comparison to other sequences	yes	yes
Sartoretti et al*.* [15]	2022	55 patients	1.5 T, Philips Achieva and Ingenia	yes	3D T2 TSE SAG vs 2D T1 & T2 TSE SAG	no	no
Lee et al. [3]	2015	42 patients	3.0 T, Siemens Magnetom Verio	yes	3D T2 SPACE SAG vs 2D T2 TSE SAG, AX & COR	yes	yes
Yamada et al*.* [5]	2015	40 patients + 20 controls	3.0 T Philips, Achieva	yes	3D FFE with selective excitation (“Proset Myelo”) vs. 2D TSE T1 SAG	yes	yes, 40 cases
Sung et al*.* [7]	2017	37 patients	3.0 T, Siemens Magnetom Verio	yes	3D T2 SPACE vs 2D TSE (T1 SAG, T2 SAG & T2 AX with several different combinations)	yes	*no*
Bratke et al*.* [23]	2019	10 patients and 20 healthy volunteers	3.0 T, Philips Ingenia	no	T2 3D TSE (CS & SENSE) vs. 2D T2 TSE (SAG & AX)	no	no
Morita et al*.* [13]	2020	16 healthy volunteers	3.0 T, Philips Ingenia CX, R5.4	yes	3D T2 VISTA COR with SENSE vs 3D T2 VISTA COR with Hybrid-CS	no	no
Kong et al*.* [2]	2021	90 patients	3 T, Siemens Trio Tim	yes	3D T2 SPACE COR (CE SPAIR) vs. 2D FSE (T1 SAG, T2 SAG & T2 AX)	yes	yes

**Table 2:** Studies comparing 2D and 3D MRI sequences in cervical spine

Authors	Publication year	Sample size (cases/controls)	Field strength and MRI machine	Inter ± intraobserver (yes/no)	MRI sequences used	Diagnostic performance (sens/spesf etc.) (yes/no)	Surgical correlation (yes/no)
Meindl et al*.* [16]	2008	15 healthy volunteers	1.5 T, Siemens Avanto	yes	3D T2 SPACE SAG & AX vs 2D T2 TSE SAG & AX and T2* GRE AX	no	no
Abdulhadi et al. [26]	2014	30 patients	1.5 T, Siemens Avanto	no	3D T2 FSE SAG vs 2D T2 FSE AX	no	no
Fu et al*.* [18]	2016	48 patients	1.5 T, Siemens Esprit or Avanto 3.0 T, Siemens Verio	yes	3D T2 TSE vs 2D FSE (T1, T2 and PD)	no	no
Chokshi et al. [19]	2017	45 patients	1.5 T, Siemens Aera	yes	3D T2 SPACE SAG vs 2D T2 FSE AX & SAG	no	no
Barnaure et al*.* [20]	2022	60 patients	1.5 T, Siemens Avanto-fit or 3.0 T, Siemens Skyra-fit	yes	3D T2 SPACE SAG vs 2D T2 TSE AX & SAG	no	no
Kwon et al*.* [17]	2012	14 healthy volunteers	3.0 T, Philips Intera Achieva	yes	3D T2 VISTA SAG vs 2D T2 TSE SAG, AX & Obliques	no	no
Xiao et al*.* [30]	2015	15 healthy volunteers	3.0 T, Philips Achieva	no	3D T2 FFE AX vs 2D T2 TSE AX	no	no
Asiri et al*.* [22]	2021	8 healthy volunteers	3.T, Siemens Prisma	no	3D T2* MEDIC AX vs 2D T2* MEDIC AX	no	no
Wang et al*.* [10]	2021	45 patients	3.0 T, Siemens Skyra	yes	3D DESS vs MEDIC vs 3D SPACE	yes (N = 31)	yes
Jardon et al*.* [21	2022	41 patients	3.0 T, GE Signa Premiere	yes	3D T2 FSE SAG (AIR Recon DL) vs. 2D T2 FSE AX & SAG	no	no

## Diagnostic performance

Intuitively, one could argue that 3D MRI of the spine would offer superior diagnostic performance in the detecting recess, central and foraminal stenoses. However, only seven studies have produced these metrics, all focusing on lumbar spine. Five studies have reported diagnostic performance applying surgery as the gold standard. In 2021, Kong et al. studied 90 patients with 165 explored nerve roots assessing the central spinal, lateral recess, intraforaminal, or extraforaminal stenoses. The sensitivity, specificity, positive predictive value (PPV), negative predictive value (NPV), and accuracy for the 2D MRI were 78.3%, 72.7%, 94.9%, 34.0%, and 77.6%, respectively. For 3D T2-SPACE sequence, superior values were found with the sensitivity, specificity, PPV, NPV, and accuracy being 91.6%, 86.4%, 97.8%, 61.3%, and 90.9%, respectively [2]. Similarly, in 2015, Lee et al. evaluated 42 patients with surgical correlation finding comparable sensitivities between 3 and 2D sequences for foraminal stenosis (78.9% vs. 78.9%), spinal stenosis (100% vs. 100%), and recess stenosis (92.9% vs. 81.8%) [3]. Three more studies have described the diagnostic performance of 3D sequences for L5 nerves only: Hashimoto et al. (2021) conducted a study with 54 patients applying only a T1-weighted 3D sequence with surgical correlation to evaluate foraminal stenosis of L5 roots;, they reported a rather low sensitivity of 72.6% and specificity of 66.3% [4]. In a study with 40 patients with L5/S1 level intra- or extraforaminal stenoses, Yamada et al. (2015) reported superior sensitivity for 3D MRI (90%) vs. 2D MRI (63%), but similar specificities (98% vs. 100%), respectively; the overall AUC was higher for 3D MRI (0.99) vs. 2D MRI (0.94) (*p* < 0.05) [5]. Nemoto et al. studied 15 patients with L5 radiculopathy with surgical correlation. For 2D FSE T1 sagittal and 2D FSE T2 axial images the sensitivities were between 26–60%, specificities 86–91%, PPVs 57–64%, and NPVs 74–83%; for the 3D FIESTA sequence in axial, sagittal and coronal views superior metrics were observed with the respective values being 60–100%, 94–97%, 82–94%, and 85–100% [6].

One study used the radicular leg pain as the reference standard to evaluate the performance of 2D TSE, 3D TSE or various combinations of these sequences in 37 patients (78 nerve roots in total). Somewhat surprisingly, the study observer similar sensitivities (range 81–94%), specificities (range 54–67%) and AUCs (range 0.764–0.843) across the different sequence combinations, with no statistical significance between their overall accuracy [7]. Lastly, two studies used only MRI sequences as the gold standard. With 250 patients, Sayah et al. (2016) examined the diagnostic performance of standard 2D and 3D protocol. The combination of these all sequences was considered as the gold standard. 3D and 2D protocols’ sensitivities were 68.7% and 66.3% for disk herniation, 85.2% and 81.5% for central canal stenosis, 82.9% and 69.1% for lateral recess stenosis, and 76.9% and 69.7% for foraminal stenosis, respectively [8]. In an emergency room setup, Koontz et al. (2017) examined the value of sole T2 SPACE fs sequence against conventional lumbar MRI protocol. For various pathologies, high specificities of 89–100% (CIs 82–100%) were seen coupled with high sensitivities of 100% (CIs 3–100%) excluding nerve root impingement, discs, hematoma and metastases that yielded only sensitivities of 0–69% (CIs 0–99%) [9]. Regarding the cervical spine, only one study briefly reported the diagnostic performance of a single sequence against surgical correlation: Wang et al. (2021) showed that 3D-DESS sequence in 31 patients with cervical spondylosis were very comparable with surgical findings – the agreement rate was 93.5% [10].

## Diagnostic agreement and reliability

Nowadays, diagnostic methods must be not only accurate but also reproducible. There are several studies addressing agreement (how close the results of the repeated measurements are) and reliability (ability of the scores to distinguish between subjects) for lumbar spine between 2 and 3D T2 sequences. The obtained results and used methods have been heterogeneous and sometimes even confusing.

In a study by Kong et al. (2021), interrater reliability for 2D FSE and 3D SPACE was excellent (k = 0.868 and k = 0.947, respectively). However, intermethod reliability for central canal stenosis, recess stenosis, intraforaminal and extraforaminal stenosis showed low to moderate kappa-values ranging from 0.276 to 0.571 [2]. Sung et al. (2017) demonstrated was almost perfect interrater reliability for 2D T2 and 3D SPACE sequences in the evaluation of all causes of nerve root compromise (k = 0.88–0.97) [7]. Hossein et al. (2018) reported substantial interrater reliability for pathologic indexes (degeneration, herniation, stenosis) (k = 0.603 and k = 0.733) and visibility scores in different anatomical structures (k = 0.630–0.955) for 2D TSE and 3D SPACE, respectively. Intermethod reliability for two radiologists was also substantial (k = 0.679 and k = 0.896) [11]. High intermethod, interrater and intrarater agreement in 2D T2 FSE and 3D T2 TSE concerning degenerative changes has been reported by others [12].

Morita et al. (2020) showed moderate to substantial interrater reliability in various image quality parametersin a study comparing 3D T2 VISTA with SENSE or faster hybrid compressed sensing (hybrid CS) in healthy subjects [13]. Swami et al. (2016) reported both interrater and intermethod reliability for lumbar central canal stenosis ranging from substantial to near perfect on both 3D SPACE and 2D MRI sequences, and superior interrater reliability for 3D [14]. Lee et al. reported better interrater reliability for 3D SPACE TSE compared to 2D T2 TSE for foraminal stenosis (k = 0849 vs 0.451), central stenosis (k = 0.809 vs k = 0.503) and recess stenosis (k = 0.681 vs 0.429) [3]. Nemoto et al. (2014) reported interrater reliability in evaluating L5 nerve stenosis as substantial for axial 2D T2 TSE (k = 0.735) and for axial and sagittal 3D FIESTA (k = 0.733 and k = 0.750 respectively) and excellent for coronal 3D FIESTA (k = 0.953) [6]. Similarly, in grading lumbar recess and foraminal stenosis, interrater reliability was near perfect for both T2 2D and 3D TSE (k = 0.823–0.945), whereas the intermethod reliability was only moderate (k = 0.543–0.577) [15]. With emergency room patients, high interrater agreement for severe conditions using 3D T2 space imaging has been also shown [9].

The literature on the cervical spine is more limited. However, in healthy individuals, interrater reliability on visibility of the evaluated structures has been reported substantial or near perfect in T2 2D and 3D sequences [16, 17]. Fu et al. (2016) assessed degenerative changes, stenosis and herniation, reporting high absolute agreement rates for both 3D and 2D sequences (75.9% vs. 75.7%), with moderate interrater reliability (k = 0.43) and high overall intermethod agreement (80.7%) [18]. In image quality study, interrater reliability was reported only slight or fair for both 3D and 2D T2 sequences [19]. Barnaure et al. (2022) found moderate to substantial interrater reliability for grading foraminal stenosis with both 3D and 2D sequences, though slightly lower reliability was observed for a specific level at C7/Th1 in the 3D sequence [20]. Interrater reliability for foraminal stenosis was substantial for 2D T2 sequences (k = 0.76) and excellent for 3D T2 sequence reconstructed with deep-learning-based algorithm (k = 0.81). Furthermore, reliability was excellent for both sequences (k = 0.83–0.85) for central stenosis [21]. The only reliability study with just ICC and no kappa values reported high (r = 0.962) consistency between readers in 3D DESS, MEDIC and 3D SPACE [10].

All in all, for both cervical and for lumbar spine, there seems to be a high degree of agreement between both visibility and diagnostic findings on 3D TSE and 2D FSE. In addition, intra- and interrater reliability has mostly been at least as good or better for 3D sequences.

## Imaging techniques and comparative analyses

The 3D sequences commonly discussed in the literature include fast/turbo spin echo based SPACE, VISTA and general FSE/TSE (Tables 1 and 2), while Wang et al. (2021) and Asiri et al. (2021) included 3D DESS and 3D MEDIC sequences in their studies[10, 22]. These 3D T2 imaging sequences are typically performed in the coronal and sagittal planes with isotropic resolutions ranging from 0.6 to 1.0 mm, with Asiri et al. (2021) highlighting the usefulness of isotropic 0.3 mm voxel size (3D MEDIC) [22]. In contrast, 2D sequences used for comparison varied widely, from singular T1 or T2 weighted images to whole protocols including variations of T2, T1, and PD weighted sequences (Tables 1 and 2).

The reported imaging times for the 3D sequences ranged from approximately from three to eight minutes. Notably, both Swami et al. (2016) and Sayah et al. (2016) reported similar image quality with rapid protocols using 3D SPACE (five-to-nine-minute scan times) and routine TSE protocols with total scan time of 20 to 27 min, respectively [8, 14]. Most 3D sequences were accelerated (most commonly SENSE or compressed sensing (CS)) with varying factors. Morita et al. (2020) reported locally increased CNR and SNR with reduced the scan time of 3D T2 VISTA using a hybrid CS acceleration method compared to SENSE [13], while Bratke et al. (2019) found adequate image quality with acceleration factors of 2.5 (SENSE) and 4.5 (CS) in 3D SPACE sequences [23].

Evaluation of SNR or CNR between two different sequences without a quantitative reference or precise choice of sequence parameters is challenging. This can also be concluded from the gathered literature (Tables 1 and 2) as only a few articles included quantitative evaluations of image quality with 3D sequences [16, 17, 22–26]. The reported SNR and CNR values from 3D sequences were comparable or higher than those reported from comparable 2D sequences; for instance, Kwon et al. (2012) reported improved SNR with 3D VISTA compared to conventional 2D TSE sequences [17]. Wang et al. (2021) compared the CNR from 3D DESS, MEDIC and SPACE sequences, and found the 3D DESS to be superior when imaging the nerve roots, while 3D SPACE performed best in imaging the cerebrospinal fluid [10]. Moreover, advancements in deep learning reconstruction methods have led to improved image quality with 3D sequences [21, 27].

## Discussion

Here we have reviewed systematically the relevant scientific literature starting from the twenty-first century regarding the clinical utility of 3D MRI in the assessment of stenoses of the spine. In general, a lot of heterogeneity exists among the studies conducted on this subject including the study setups, imaging techniques, reference standards, assessment of diagnostic performance and reliability analyses.

**Fig. 1:**
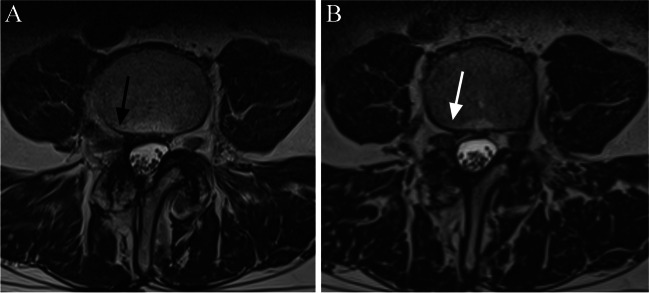
Comparison of 2D and 3D MRI sequences with corresponding axial images at the lumbar level of L3/4. In the conventional 2D T2 TSE axial image the degree of foraminal stenosis remains unclear (black arrow) due to scarring from previous surgery and partial volume effect (**A**). In the 3D T2 TSE (SPACE) image, no stenosis is detected (white arrow) (**B**)

**Fig. 2:**
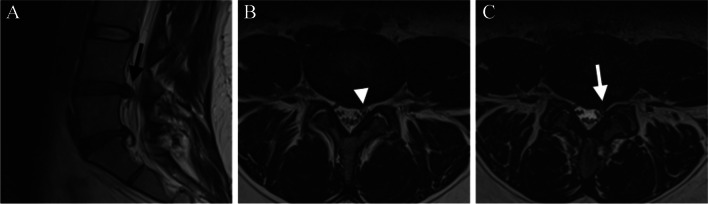
Evaluation of lumbar recess stenosis on 2D and 3D MRI sequences. A typical L4/5 disc herniation is seen on conventional 2D T2-weighted sagittal MR image (**A**). In the routine 2D T2 TSE axial image no recess stenosis is visible (white arrowhead) (**B**), whereas in the 3D T2 TSE (SPACE) image a definite stenosis is seen in the left lateral recess (white arrow) (**C**)

**Fig. 3:**
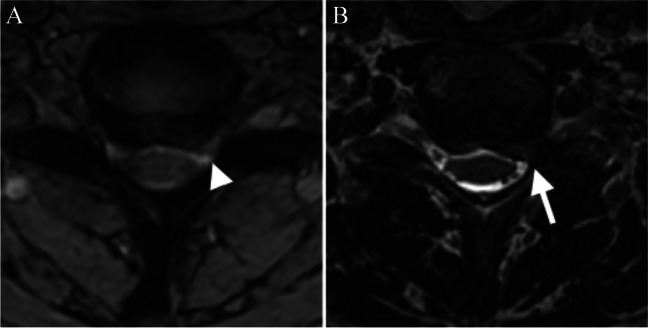
Comparison of 2D and 3D MRI sequences with corresponding axial images at the cervical level of C6/7. In the conventional gradient echo axial image, there seems to be only mild stenosis on the left side (white arrowhead) (**A**). In the 3D T2 TSE (SPACE) image, severe stenosis is detected (white arrow) (**B**)

**Fig. 4:**
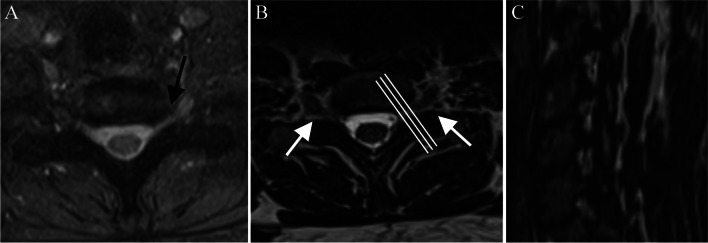
Assessment of cervical foraminal stenosis on 2D and 3D MRI sequences. Conventional 2D T2-weighted GRE sequence (MEDIC) shows moderate stenosis of the left C8 foramen (black arrow) probably due to partial volume effect (**A**), since in 3D T2 weighted TSE (SPACE), the C8 foramina are wide and symmetrical (white arrows) (**B**). Another advantage of 3D sequence is the ability to visualize nerve canals in sagittal oblique reformats (**C**) (as shown by the perpendicular white lines in B)

Lumbar and cervical MRI examinations are the most common MRI scans in everyday clinical workflow to evaluate radiculating extremity pain, suspected central stenosis or prolonged back pain. Historically, the lumbar MRI usually consists of several routine 2D sequences (T2 & T1 weighted sagittal, T2 weighted axial and STIR coronal planes), and the cervical MRI includes 2D T1 & T2 weighted sequences in sagittal plane and T2 weighted FSE or GRE sequences in axial plane. However, to date, no specific guidelines for the exact composition of lumbar or cervical MRI protocol exists. Although the use of 3D lumbar and cervical spine MRI sequences has transpired since the 2010s, the current literature offers no answer, which 3D sequence is the best or most optimal to image spine and associated pathologies. Based on this current literature review, it seems that 3D sequences offer superior overall evaluation of recess stenoses [3, 8] and increased sensitivity to detect foraminal (both intra- and extra-) stenoses at least in lumbar level [2, 6–8]. These observations have also been confirmed at our institution intuitively (Figs. 1, 2, 3 and 4). Although the evaluation of central canal stenosis seems to be equivalent in both 2D and 3D MRI techniques, it could be argued that 3D sequences provide some aid in the respect of accurate slice orientation; this phenomenon was already confirmed in the year 2012 by Henderson and colleagues, who showed that 3D MRI approach was superior in the measuring and grading of the lumbar central canal stenosis [28].

The main focus of cervical MRI is usually in diagnosing stenosis. However, demonstration of abnormal signal in the cord (i.e. spondylotic myelopathy) is also important, especially since it may serve as a prognostic tool in aiding surgeons with clinical decision making [29]. Traditionally, it has been thought that gradient based T2 sequences are superior or even mandatory in diagnostic work-up of cord pathology. Indeed, delineation of spinal cord structures and CNR were better in gradient based 3D FFE sequence than in 2D TSE sequence in a study with healthy volunteers [30]. However, also 3D T2 FSE sequences have been shown feasible in spondylotic myelopathy, even though poorer CNR compared to conventional 2D FSE sequence was evident [18, 26]. Postoperative recovery varies greatly even with similar cord pathology on imaging. Urakawa et al. (2011) speculated that this may partly be due to inability of conventional images to distinguish individual tracts and presented 3D anisotropy contrast single-shot echo planar imaging as a solution [31]. Thus, it may well be that adequate imaging of cord pathology requires completely different methods beyond the scope of this review article.

Based on the literature it is difficult to conclude the best 3D sequence to use for imaging spinal stenosis. More specifically, quantitative comparison between 3D and 2D based sequences can be cumbersome due to the fact that the contrast, resolution and scan time all are affected by the choice of the sequence parameters (mostly, the choice of repetition time (TR), echo time (TE), number of slices etc.)), type of sequence (e.g. Spin-echo and Gradient echo based sequences; T1, T2 –weighted and inversion recovery –based sequences etc.) and the used field strength (1.5 T vs. 3 T). For example, traditional 3D spin echo (SE) sequences (e.g. SPACE, CUBE etc.), require longer acquisition time and different contrast properties than 3D gradient echo techniques (e.g. MPRAGE, MERGE etc.). The long acquisition times can be overcome by using techniques that accelerate the imaging process, for example parallel imaging or CS [13, 23]. Most of these acceleration methods are not lossless and may deteriorate the final image, however, recent studies have demonstrated that developments in deep learning reconstruction methods may help to overcome this [21, 27].

The literature also showed nonuniform voxel sizes, interpolation and varied use of reformatted image planes from the 3D images. Particularly, the use of isotropic voxel sizes is advised in 3D imaging to get uniform reformats in all three planes. Notably, most 3D sequences gathered here were imaged in either sagittal or coronal planes which require generally larger FOVs, but less slices to cover the regions of interest, while in the authors' institution, the imaging in done in axial plane. The choice of primary phase-encoding direction (i.e. the imaging plane) can affect imaging time and manifestation of imaging artefacts (wrapping, flow and motion artefacts and geometric distortion).

All in all, many challenges exist in comparing 3D and 2D sequences. Particularly, when it comes to the choice of compared imaging techniques and sequences, there seems to be notable discrepancy (e.g. comparisons between T2-weighted and T1-weighted images, between sequences imaged with scanners from different vendors, different types of sequences (SE, GRE, STIR etc.)) (Tables 1 and 2). Nevertheless, well optimized 3D sequences allow the use of higher spatial resolution, similar scan time and increased SNR and CNR when compared to 2D sequences [11, 13]. However, the largest benefit of 3D sequences is in the clinical value of the additional information provided by them and in the possibility to replace a number of 2D sequences to save time. Especially, the ability to review images in any orientation allows better visualization of stenosis and is extremely beneficial in anatomical deformities such as scoliosis [8].

## Strengths and limitations

Strengths of this review include the rigorous assessment of the literature by three academic medical experts: a neuroradiologist, a musculoskeletal radiologist and a medical physicist. Moreover, we applied the PRISMA recommendations for meticulous reporting of our findings. One limitation is that relevant articles might not have been included due to the limited number of databases used in the search or limitations in the search and screening strategy. The most obvious weakness within this systematic review is vast heterogeneity of the included studies, most importantly the lack of surgical gold standard is worrisome. Accordingly, there was no possibility of meta-analysis. Moreover, the fact that no studies on thoracic spine existed in the literature remains as minor weakness.

## Conclusions

In conclusion, the literature of the 3D MRI assessment of spinal stenoses is largely heterogeneous with varying MRI protocols and diagnostic results. Generally, 3D sequences offer similar or superior detection of stenoses with high reliability explained by the better visualization of anatomic structures. Ultimately, the benefit of 3D MRI seems to be the better evaluation of recess stenoses which supports the clinical implementation of these sequences into everyday workflow.
